# Modulation of the effects of a cholesterol-supplemented high-fat diet by aryl hydrocarbon receptor (AHR) activation and/or tryptophan reduction in male mice

**DOI:** 10.1016/j.toxrep.2025.102083

**Published:** 2025-07-07

**Authors:** Avinash Bathina, Janne Hakanen, Atso Raasmaja, Jere Lindén, Laura Mairinoja, Suraj Unniappan, Lars Pettersson, Raimo Pohjanvirta

**Affiliations:** a Department of Food Hygiene and Environmental Health, Faculty of Veterinary Medicine, University of Helsinki, Helsinki, Finland; b Division of Pharmacology and Pharmacotherapy, Faculty of Pharmacy, University of Helsinki, Finland; c Finnish Center for Laboratory Animal Pathology, HiLIFE-Helsinki Institute of Life Science, University of Helsinki, Helsinki, Finland; d Research Centre for Integrative Physiology and Pharmacology and Turku Center for Disease Modeling, Institute of Biomedicine, University of Turku, Turku, Finland; e Laboratory of Integrative Neuroendocrinology, Department of Veterinary Biomedical Sciences, Western College of Veterinary Medicine, University of Saskatchewan, Saskatoon, Saskatchewan, Canada; f Immunahr AB, Lund, Sweden

**Keywords:** Aryl hydrocarbon receptor, Strain differences, Tryptophan, High-fat diet, IMA-08401, Steatosis, CLAMS

## Abstract

Aryl hydrocarbon receptor (AHR) is a ligand-activated transcription factor whose role in energy metabolism is obscure. Most of its physiological ligands are derived from tryptophan (TRP). Here, fifty male C57BL/6JRccHsd mice were assigned to one of five feeding groups, control diet (CD), high-fat diet (HFD; 45 % of energy from fat), HFD with only 70 % of the regular TRP concentration (HFDtrp), HFD supplemented with a weakly toxic AHR agonist C2 (HFDc2), or HFDtrp with C2 (HFDtrp-c2). All diets contained 2 % cholesterol and were fed for 18 weeks. On weeks 14–16, the mice were tested for gas exchange and locomotor activity, and on weeks 15–17 for glucose tolerance (GTT) and insulin sensitivity (ITT). At termination, tissue samples were collected for biochemical and AI-assisted histological analyses. Body weight gain (BWG) was only 28–38 % higher in the HFD groups than in the CD group, but the HFD-fed mice accumulated 43–61 % more fat. Calorie intake was greater in the two low-TRP groups than in the two other HFD groups, while BWG remained similar. C2 induced *Cyp1a1* expression (an index of AHR activity) in all tissues examined and increased the ratio of micro-/macrosteatosis in the liver. The HFDs tended to reduce insulin sensitivity, CO_2_ production, and the ability to respond appropriately to a low-temperature challenge. These findings suggest that the effects of AHR activity modulation on energy balance are strongly context-dependent. A sensitive response to long-term AHR activation appears to be elevated micro-/macrosteatosis ratio in the liver when exposed to HFD.

## Highlights

• 50 male C57BL/6JRccHsd mice were fed for 4.5 months with one of five diets.
• High-fat diets (HFDs) increased calorie intake & body fat more than body weight (BW).
• HFD with low tryptophan augmented calorie intake vs. other HFDs but not BW gain.
• HFD with the AHR agonist C2 elevated hepatic micro-/macrosteatosis ratio.
• Microsteatosis showed a positive correlation with hepatic inflammatory foci.

## Graphical Abstract

**Figure:**
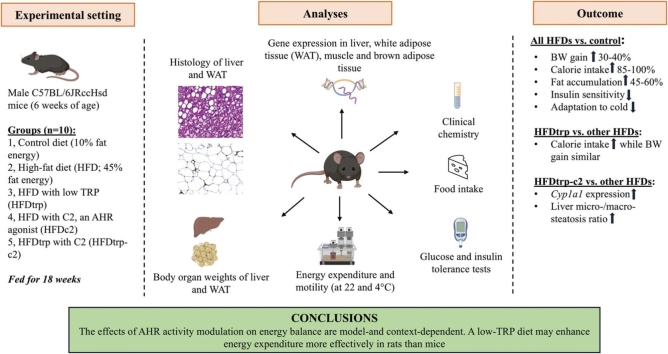

## 1 Introduction

Obesity is a global health problem and a major risk factor for a number of secondary ailments including cardiovascular diseases, type 2 diabetes, non-alcoholic hepatic steatosis, and cancer [1], [2]. Consumption of high-calorie food, sedentary lifestyle, genetics, and lack of exercise are some of the main causes of obesity [3]. Obesity develops gradually if energy intake persistently exceeds energy expenditure. While a substantial amount of information has been gathered on the endocrine and neurochemical factors as well as central structures regulating food intake, much less is known about the regulation of energy expenditure. Recent studies have suggested that one player involved in energy expenditure might be the aryl hydrocarbon receptor (AHR).

AHR is a ligand-activated nuclear receptor which was originally found as the mediator of toxicity of dioxins and polycyclic aromatic hydrocarbons, the most potent of which is 2, 3, 7, 8-tetrachlorodibenzo-*p*-dioxin (TCDD) [4]. AHR controls the transcription of hundreds of genes, the best-known of which are related to the metabolism of xenobiotics [5]. One of the most sensitive markers of canonical AHR activation is the induction of *Cyp1a1* expression [6]. During the past couple of decades it has become clear that AHR is also a key regulator of numerous physiological cellular processes, ranging from cell proliferation and apoptosis through liver, reproductive organ, immune system and blood vessel development to the maintenance of barrier tissue integrity [7], [8].

Regarding energy balance, studies in the obesity-prone C57BL/6J mice have indicated that for feeding on a Western diet or a high-fat diet (HFD) to result in obesity, the participation of AHR is required. In addition to conferring resistance to obesity, global AHR deficiency was reported to protect mice against other HFD-induced disorders of the metabolic syndrome including visceral fat accumulation and inflammation, insulin resistance, glucose intolerance, and steatohepatitis; interestingly, body weight gain on a standard diet was not modified [9]. Similar findings were obtained when AHR activity was inhibited in mice with an AHR antagonist [10], [11], [12]. Although the principal target tissue remained uncertain, AHR-knockout (AHRKO) mice exhibited increased energy expenditure in association with induction of thermogenic genes in the brown adipose tissue (BAT) and induction of β-oxidation genes in the skeletal muscle [9].

In further support to a regulatory role of AHR in energy metabolism, serum AHR ligand activities were found to correlate with obesity and the metabolic syndrome in humans [13], and a long-term treatment with a subtoxic dose of TCDD exacerbated body weight gain on HFD in mice [14], [15]. While TCDD is the most potent AHR agonist among environmental contaminants, metabolites of the strictly essential amino acid tryptophan (TRP) present in the diet, produced by microbiota, or formed endogenously have been identified as the major physiological activators of AHR [16]. Two prominent metabolites are kynurenine and 6-formylindolo[3,2-*b*]carbazole (FICZ) [17]. In line with the influence of subtoxic TCDD exposure, dietary treatment of mice with kynurenine enhanced body weight gain and induced fatty liver and hyperglycemia. In this case, however, the mice were fed with a low-fat (10 % of energy from fat) diet [18].

The available evidence thus appears to point to prevention or inhibition of obesity and the metabolic syndrome with impaired AHR function and, conversely, their aggravation with induced AHR activity. However, the picture is still far from clear, because some studies have reported conflicting outcomes. For example, experimental periodontitis was shown to lead to AHR inactivation in serum, feces and proximal small intestine of C57BL/6N mice accompanied by accelerated body weight gain, increased fat mass, and insulin resistance. Restoration of AHR activity by FICZ supplementation slowed the rate of weight gain, mitigated fat accumulation and promoted insulin sensitivity [19]. In another study, a synthetic pelargonidin (Mt-P) proved to transactivate AHR and to attenuate body weight gain, intestinal and hepatic inflammation, and glucose intolerance, yet without alleviating liver steatosis in C57BL/6 mice on HFD supplemented with fructose. These effects were abrogated by ablation of the *Ahr* gene [20]. Of note, in this study global AHRKO failed to protect the HFD + fructose-fed mice from obesity and hepatic steatosis. In still another study, administration of indole-3-carbinol, which is converted to the highly potent AHR agonist indole[3,2-b]carbazole in the acidic environment in the stomach, inhibited body weight gain [21]. Finally, when administered once a week for 12 weeks, the endogenous AHR agonist FICZ increased glucose tolerance and insulin sensitivity and attenuated hepatosteatosis in HFD-fed mice, and these effects were recapitulated by supplementation of mice with a Lactobacillus strain that highly produces AHR ligands [22]. These authors further reported that in humans, the metabolic syndrome was associated with lowered AHR activity in the feces.

The present study was thus constructed to shed further light on the influence of AHR on energy metabolism. We were especially interested in seeing whether a long-term activation of AHR signaling with a highly potent but meagerly toxic novel AHR agonist, IMA-08401 (C2) [23], would be able to modify the effects of HFD on energy balance. In addition, as it was previously reported that a 30 % reduction in dietary TRP concentration induced thermogenesis without altering body composition in obesity-prone rats fed on HFD [24], we surmised that this could be due to diminished AHR activity. Therefore, we included in the study protocol a HFD diet (HFDtrp) with 30 % lower TRP concentration than that in the neat HFD. So far, most feeding studies have been carried out in C57BL/6J mice because of their high susceptibility to diet-induced metabolic disorders. This susceptibility stems from a mutation resulting in the absence of the nicotinamide nucleotide transhydrogenase (*Nnt*) gene product. Consequently, their glucose tolerance is defective, insulin secretion impaired, and they exhibit mitochondrial redox abnormalities [25], [26]. To ascertain whether AHR activity alterations could modify energy metabolism in a physiologically more normal animal model, in the present study we employed C57BL/6JRccHsd mice. This substrain is derived from C57BL/6J but branched from it in 1973 and therefore does not contain the *Nnt* gene mutation, which occurred between 1976 and 1984 [27].

## 2 Materials and methods

### 2.1 Animals and their husbandry

Fifty male C57BL/6JRccHsd mice (4 weeks old) were purchased from Harlan (Netherlands). After arrival, the mice were acclimatized to the ambient conditions for two weeks before the onset of the study. They were housed singly in individually ventilated cages (Sealsafe IVC Blue Line, Techniplast, West Chester, PA, the USA) throughout the course of the experiment since male C57BL/6 mice tend to fight readily when housed in groups. Recent behavioral and hormonal analyses have shown that individual housing should not be too stressful to mice [28]. Moreover, the cages were suspended in a single rack, and they had transparent walls. This allowed visual and auditory contact among the mice. The mice were maintained on a 12-h light/dark cycle. In the morning, the lights were turned on at 6 a.m., and during the night, a dim red light was sustained. The cage floor was covered with aspen-wood bedding (Tapvei, Estonia). Each cage was provided with a transparent plastic tube, nesting material, and a wooden chew block (aspen wood, Tapvei, Estonia). Commercial pelleted rodent chow (Teklad Global 16 % Protein Rodent Diet, Teklad Diets, Madison WI, the USA) and filtered UV-irradiated tap water were available ad libitum (the chow during the 2-week acclimation period only). The animal room was air-conditioned and maintained at a temperature of 22 ± 1 °C and relative humidity of 38–75 % (typically 50 %). All experiments were authorized by the National Animal Experiment Board in Finland (Eläinkoelautakunta, ELLA, license code ESAVI/45477/2019). All procedures were conducted in accordance with Directive 2010/63/EU of the European Parliament and Council.

### 2.2 Chemicals

The test compound C2 (IMA-08401; N-acetyl-N-phenyl-4-acetoxy-5-chloro-1,2-dihydro-1-methyl-2-oxo-quinoline-3-carboxamide; CAS: 1373260-17-3) was synthesized as described earlier [29]. C2 stock solutions were prepared by mixing with polyethylene glycol 400 (PEG-400) and heating at 80 °C for 60 min.

### 2.3 Experimental design

**Fig. 1:**
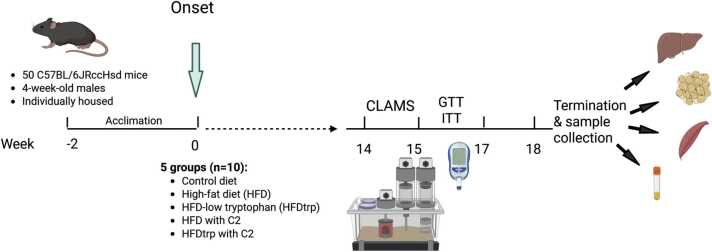
Schematic representation of the timeline and methods applied in the study. Created in BioRender. Pohjanvirta, R. (2024) https://BioRender.com/r53s977.

The experimental design is outlined in Fig. 1. The mice were randomly distributed into five feeding groups (10/group; GraphPad quick cals randomization method) after which any major deviations in mean body weights were adjusted. These five groups were the following (the compositions of the diets are in Supplemental Table S1): Control diet (CD) (10 % of energy from fat); neat high-fat diet (HFDn) (45 % of energy from fat); HFDn with a 30 % reduction in TRP concentration (HFDtrp); HFDn enriched with (3.7 mg/kg feed; ∼ 1 mg/kg body weight/day) C2, a potent but only weakly toxic, short-acting AHR agonist (HFDc2) [23]; and HFDtrp-c2. All these diets contained 2 % cholesterol. C2 was dissolved in PEG400 as described earlier [23]. The CD (A22030901), HFDn (A22030301) and HFDtrp (A22022801) diets were purchased from Research Diets (New Brunswick, NJ, the USA). The energy content was 3.7 kcal/g for the CD diet and 4.6 kcal/g for the HFDn and HFDtrp diets. All ingredients (including amino acids, minerals and fiber [cellulose]) were matched per kcal, based on published data showing that rodents adjust their feed consumption for feed energy concentration [30], [31], [32].

The diets were provided daily to the mice on petri dishes as a fresh slurry comprising 5 g of the powdered diet and 1 ml of water mixed with either C2-PEG400 or PEG400. Food consumption was measured every 24 h. The mice were weighed every three days for 18 weeks. During weeks 14–16, the mice, 10 at a time, were tested in a comprehensive laboratory animal monitoring system (CLAMS; Columbus Instruments, Columbus, OH, the USA) for their exchange of oxygen and carbon dioxide and for locomotor activity. On weeks 15–17, one week apart, the mice were subjected to glucose and insulin tolerance tests (GTT and ITT). At the end of week 18, the mice were euthanized by CO_2_ asphyxiation followed by cardiac exsanguination from the right ventricle. The liver, epididymal fat pad (epiWAT) and the interscapular brown adipose tissue (BAT) were weighed. Samples of these as well as skeletal muscle (from the back of the thigh) along with heparinized plasma (also containing protease inhibitors and dipeptidyl peptidase IV) were snap-frozen in liquid nitrogen and then stored at − 80 °C until analysis. Moreover, liver and epiWAT tissue samples were further taken into 10 % formalin for histological evaluation.

### 2.4 RNA isolation and RT-qPCR

Total RNA was extracted from the frozen liver, BAT, epiWAT, and muscle samples, reverse-transcribed to cDNA, and select cDNAs quantified by real-time PCR as described in detail earlier [23] with the small exception that in addition to the RotorGene 3000 instrument, also Rotor-Gene Q (Qiagen, Hilden, Germany) was used to determine the mRNA abundances of the metabolic enzyme genes *Cyp1a1*, *Ucp1*, *Ucp2*, *Cd36, Fgf21, Fasn*, and *Ppara*. Primer sequences are provided in Supplemental Table S2.

### 2.5 Glucose and insulin tolerance tests (GTT and ITT, respectively)

Mice were fasted for 6 h and weighed prior to both GTT and ITT. In the GTT, 20 % glucose was injected i.p. at 75 ml/kg. In the ITT, the mice were given human insulin (0.75 U/kg) instead of the glucose. Blood glucose concentrations were measured at 0, 15, 30, 60, and 120 min with a glucometer (Aveo Blood Glucose Monitoring System; Medical Technology and Devices Germany GmbH, Frankfurt am Main, Germany). The first blood sample was collected by cutting the tip of the tail (1–2 mm) with a surgical blade to obtain a sufficient blood droplet (∼ 5 µL). Subsequent blood samples were obtained by removing the clot.

### 2.6 Histology

Tissue samples for histological evaluation were obtained from the left lateral lobe of the liver and from the epididymal white adipose tissue (epiWAT). Formalin-fixed samples were embedded in paraffin, sectioned at 4 μm thickness, and stained with hematoxylin-eosin (HE) to assess liver steatosis, inflammation, and epiWAT adipocyte size. Crown-like structures (CLS) in epiWAT (indices of inflammation) were detected with anti-rabbit ionized calcium-binding adaptor molecule 1 (Iba-1) immunohistochemistry: The adipose tissue sections were first incubated for 20 min at 99 °C in 10 mM citrate buffer (pH 6) for antigen retrieval. Primary Iba-1 antibody (CAT: 019-19741, FujiFilm, Valhalla, NY, the USA; dilution 1:500) was incubated at RT for 60 min, and Vectastain ABC-HRP kit (Vector Laboratories, Burlingham, CA, the USA) with biotinylated anti-rabbit antibody (1:200) and DAP substrate was used for primary antibody detection. The liver slides were scanned into whole slide images (WSIs) using a Pannoramic 1000 Flash digital slide scanner (3DHISTECH, Budapest, Hungary) and the adipose tissue slides using a Pannoramic 250 Flash III scanner, both employing a × 20 objective resulting in a resolution of 0.24 µm/pixel. All histological analyses were conducted blinded to the treatment groups.

Microvesicular and macrovesicular liver steatosis were quantified in WSIs using a deep learning-based model developed by Mairinoja et al. [33] in Aiforia Create 5.2, an image analysis cloud platform (Aiforia Technologies, Turku, Finland). In addition, other non-alcoholic fatty liver disease (NAFLD)–related histological alterations (lobular inflammation and hepatocellular ballooning) were scored using a system developed for humans [34]. The veterinary pathologist (J.L.) scoring the liver findings also estimated the percentage areas of microvesicular and macrovesicular steatosis, and the Pearson correlation coefficients between the model and the pathologist were 0.80 (p < 0.0001) in microvesicular steatosis and 0.77 (p < 0.0001) in macrovesicular steatosis. The human four-tier (0–3) scoring of the lobular inflammation based on the estimation of the number of inflammatory foci per 200 × microscope field [34] was too coarse for the mouse model and the number of lobular inflammatory foci was thus directly counted in the HE-stained liver WSIs using QuPath 0.4.3 [35]. The counts were expressed as an average number of inflammatory foci per 200 × microscope field to collate mouse and human data. The total analysis area per sample varied from 49 to 111 E6 μm^2^.

Adipocyte size was measured in the HE-stained WSIs using QuPath 0.4.3 [35], following the method devised by Palomäki et al. [36]. The total analysis area per sample varied from 13 to 43 E6 μm^2^, and the number of analyzed adipocytes per sample from 1500 to 2000 cells. The number of CLS [37] in the epiWAT was manually counted in Iba-1-stained WSIs using the QuPath 0.4.3 Point tool in pre-annotated count areas, generally consisting of whole sections excluding shattered or holed regions, and normalized to count area sizes, which varied from 28 to 126 E6 μm^2^ per sample.

### 2.7 CLAMS

CLAMS was used to determine several metabolic parameters simultaneously. Oxygen consumption (VO_2_) and CO_2_ production (VCO_2_) were measured for each mouse once every 14 min for 24 h. Simultaneous locomotor activity was assessed by infrared beam interruption. During the CLAMS procedure, the mice were individually housed in 10 respiratory chambers. The mice had free access to food and water during the measurements and were acclimatized to the CLAMS environment for 24 h at 22 °C before data collection. On the third day, the CLAMS temperature was decreased to + 4 °C and the same measurements were taken for another 24 h. The respiratory exchange ratio (RER) was measured as the ratio of VCO_2_/VO_2_. The energy expenditure or heat was measured using the equation: calorific value = VO_2_ × (3.815 + 1.232 × RER) [24], and expressed as kilocalories per hour, with data collection and processing done by Oxymax CLAMS version 5.0. Food and water consumption was measured daily. After the CLAMS recordings, the mice were placed back in their regular cages for a week to recover before GTT and ITT.

### 2.8 Clinical chemistry

Clinical chemistry analyses on plasma samples were carried out at the Central Laboratory of the Department of Equine and Small Animal Medicine, University of Helsinki. Enzymatic methods were used for the determination of free fatty acids (NEFA) and D-3-hydroxybutyrate (RANBUT [3-HB], both from Randox Laboratories Ltd., Crumlin, UK). The rest of the serum analytes [alanine aminotransferase (ALAT), aspartate aminotransferase (ASAT), creatinine, glucose, triglycerides, and cholesterol] were analyzed using the reagents and adaptations recommended by the manufacturer of the automatic chemistry analyzer (Konelab 30i, Thermo Fisher Scientific, Waltham, MA, the USA).

Plasma insulin levels were measured using mouse insulin enzyme-linked immunosorbent assay (ELISA) according to manufacturer’s instructions (Catalog # 80-INSMS-E01; Alpco, Salem, MA, the USA).

### 2.9 Data analysis and statistics

Body weight change and relative EpiWAT, BAT, and liver weights along with plasma biochemical analyses, histopathological findings, and RT-qPCR data were statistically analyzed by one-way analysis of variance (ANOVA) with Tukey’s post-hoc test. In the case that the assumptions of ANOVA were not met and data transformations were not able to resolve this, either Welch’s robust ANOVA with Games-Howell post-hoc test (unequal variances) or non-parametric tests (Kruskal-Wallis H or Mann-Whitney *U* test) were applied. GTT and ITT data were analyzed with two-way mixed ANOVA and, for the area under the curve [AUC] analyses, one-way ANOVA with Tukey’s post-hoc test. Body weight-dependent CLAMS data (VO_2_, VCO_2_ and heat) were evaluated by two independent methods: 1) normalizing the volume data to body weight and 2) by the analysis of covariance (ANCOVA) using body weight as the covariate as recommended by Müller et al. [38]. Additional analyses between, *e.g*., pooled HFD groups and CD were conducted by Student’s unpaired t test. Finally, correlations among the histological lesions were explored by Spearman’s rank order correlation test. The numerical data are presented as the mean ± SEM, and the level of statistical significance was set at p ≤ 0.05. All statistical analyses were carried out using the GraphPad Prism, version 8, or the Statistical Package for the Social Sciences (SPSS), version 28, software.

## 3 Results

### 3.1 Food consumption, body weight gain, and relative tissue weights

**Fig. 2:**
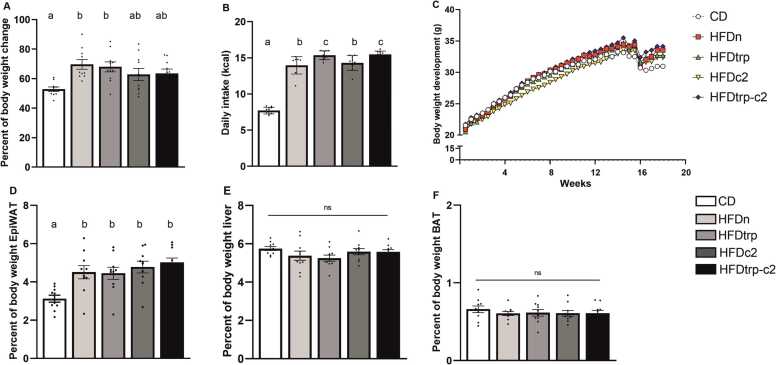
Effects of the diets on relative body weight change (A), average daily food consumption (B), body weight evolvement (C), and relative weights of the epididymal fat pad (D), liver (E), and BAT (F). Columns with non-identical letters are statistically different at the significance level (p < 0.05). Columns with “ns” denote no statistically significant difference among the groups (n = 10; mean ± SEM). In Fig. 2C, the transient decrease in body weight is due to the CLAMS analyses.

Energy intake (kcal consumed) was significantly higher in all four groups fed with the 2 % cholesterol-containing HFD (45 % of energy from fat) as compared with the CD-fed group. Interestingly, the intake of the two HFD varieties whose TRP concentration was diminished was significantly higher than that of their HFD counterparts with normal TRP concentration (Fig. 2**B**). In contrast, addition of C2 did not have any effect on food consumption. Body weight gain was higher in the HFD groups than in the CD group, but when the dietary groups were assessed individually, the difference reached significance only for the HFDn group (Figs. 2**A &** 2**C**). However, when the four HFD groups were pooled (to verify the effect of augmented energy intake), their body weight gain proved to be significantly greater than in the CD group (p = 0.003).

In relative tissue weights (measured for epiWAT, BAT and liver), the only statistically significant alterations were recorded in epiWAT, with all the HFD-fed groups showing higher values than the CD group (Fig. 2**D–F**).

### 3.2 Glucose and insulin tolerance tests

**Fig. 3:**
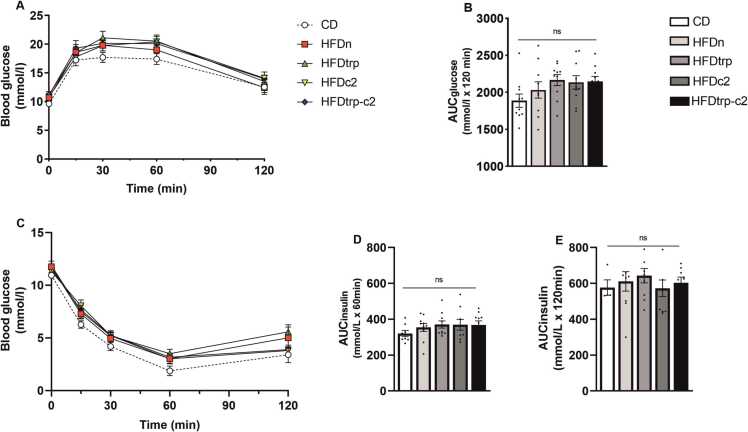
Effects of the diets on blood glucose levels in the glucose (panels A & B) and insulin (panels C–E) tolerance tests. The course of blood glucose changes over the 2-h observation time is depicted in A and C, and the respective areas under the curves in B, D and E. There were no statistically significant differences in any of the panels (in B, D & E, indicated by “ns”) (n = 10; mean ± SEM).

To examine the effects of the five diets on glucose metabolism and insulin signaling, GTT was performed on weeks 15–16 (Figs. 3**A &** 3**B**) and ITT on weeks 16–17 (Fig. 3**C–E**). In GTT, blood glucose concentration exhibited the least prominent change from its basal level in the CD group but the difference from other groups did not attain statistical significance. Nevertheless, the AUC of pooled HFDs did differ from CD’s AUC (p = 0.014). In ITT, some of the mice became so hypoglycemic by 60 min that they had to be revived with an i.p. glucose injection. Therefore, the AUC was calculated separately for 60 (Fig. 3**D**) and 120 min (Fig. 3**E**) with somewhat different group sizes. The most insulin-sensitive group proved to be again CD. Despite failing to show statistical significance when assessed individually, pooled HFD groups did differ significantly from CD at 60 min (p = 0.044), but not at 120 min.

### 3.3 CLAMS

**Fig. 4:**
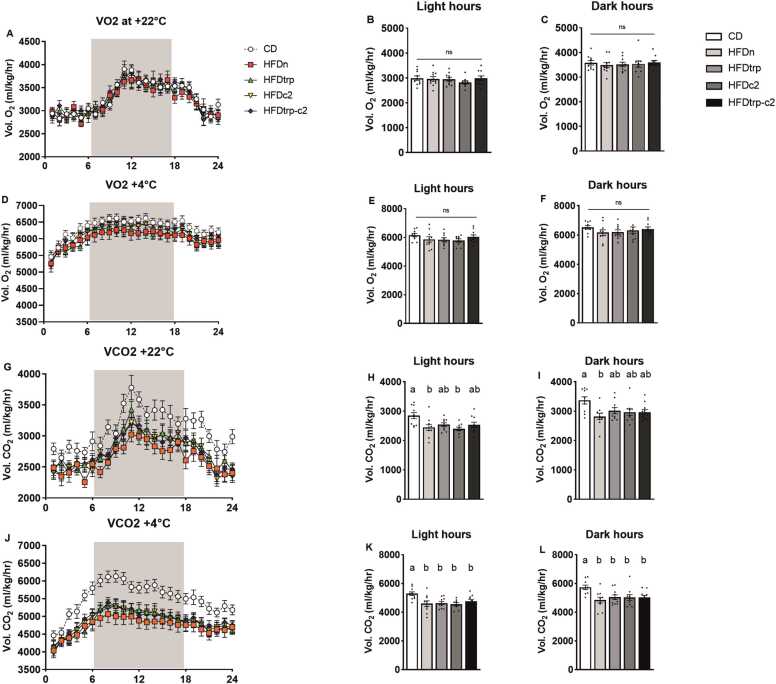
Indirect calorimetry recordings in CLAMS. Hourly mean VO_2_ consumption (A) at 22 ^ο^C, mean VO_2_ consumption during the 12 light hours (B), and mean VO_2_ consumption during the 12 (shaded) dark hours (C). Hourly mean VO_2_ consumption (D) at 4 ^ο^C, mean VO_2_ consumption during the light hours (E), and VO_2_ consumption during the dark hours (F). Hourly mean CO_2_ production (G) at 22 ^ο^C, CO_2_ production during the light hours (H) and CO_2_ production during the dark hours (I). Hourly mean CO_2_ production (J) at 4 ^ο^C, mean CO_2_ production during the light hours (K) and mean CO_2_ production during the dark hours (L). Columns with non-identical letters are statistically different at the significance level (p < 0.05). In panels B, C, E & F, “ns” denotes no significant difference among the groups (n = 10, mean ± SEM).

**Fig. 5:**
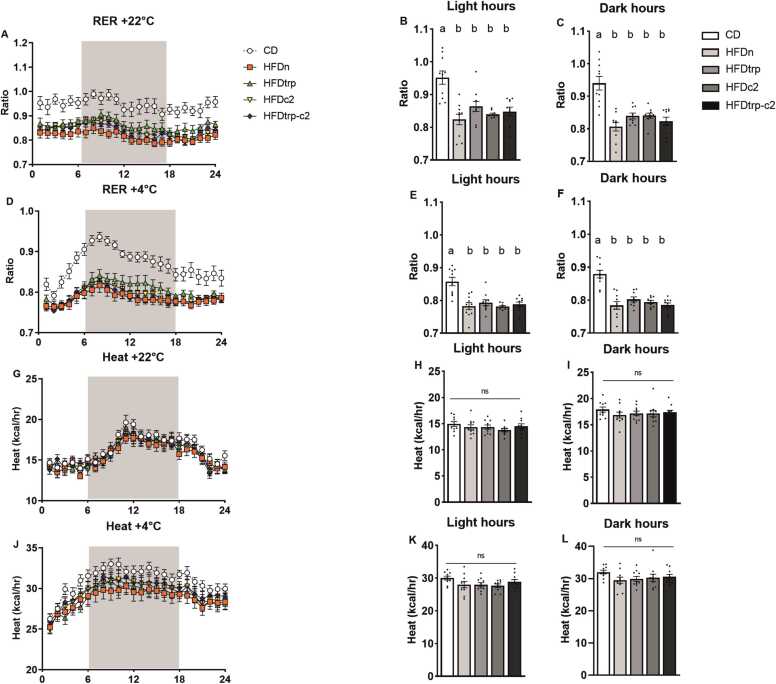
RER and heat recordings. Hourly mean RER at 22 ^ο^C (A), mean RER during the light hours (B) and during the dark hours (C). Hourly mean RER at 4 ^ο^C (D), mean RER during the light hours (E) and during the dark hours (F). Hourly mean heat at 22 ^ο^C (G), mean heat during the light hours (H) and during the dark hours (I). Hourly heat at 4 ^ο^C (J), mean heat during the light hours (K) and during the dark hours (L). Columns with non-identical letters are statistically different at the significance level (p < 0.05). In panels H, I, K & L, “ns” denotes no significant difference among the groups (n = 10, means ± SEM).

**Fig. 6:**
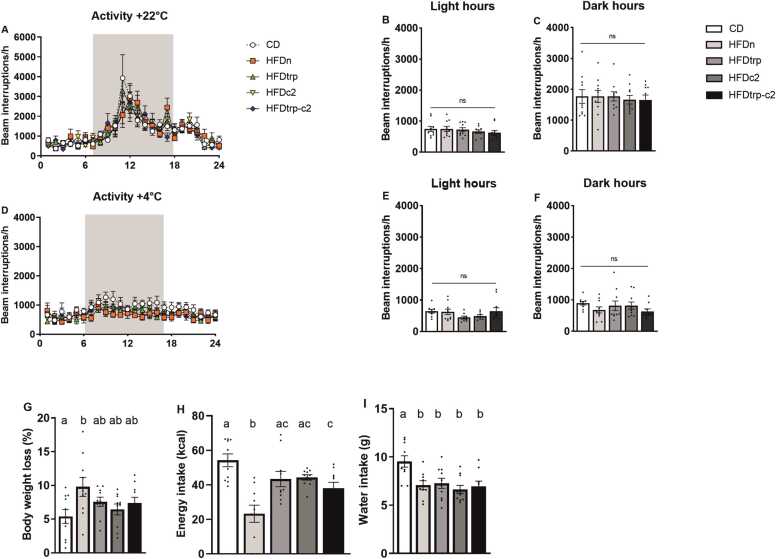
Locomotor activity of mice during the CLAMS. Hourly mean activity at 22 ^ο^C (A), mean activity during the light hours (B), and during the dark hours (C). Hourly mean activity at 4 ^ο^C (D), mean activity during the light hours (E), and during the dark hours (F). Relative body weight loss (G), calorie intake (H), and water consumption (I) during the 3-day CLAMS analysis. Columns with non-identical letters are statistically different at the significance level (p < 0.05). In panels B, C, E & F, “ns” denotes no significant difference among the groups (n = 10, means ± SEM).

Both oxygen consumption and carbon dioxide production of the mice were individually measured on an hourly basis and at two temperatures, 22 °C and 4 °C (Fig. 4**A–L**). The means and SEMs of them were also calculated for the dietary groups during the light and dark hours. Moreover, these gas volume values were used to derive the respiratory exchange ratio (RER; VCO_2_/VO_2_) (Fig. 5**A–F**) and an index of energy expenditure (heat) (Fig. 5**G–L**). Oxygen consumption did not differ significantly among the groups, whereas carbon dioxide production tended to be higher in the CD vs. the HFD groups (in particular, HFDn) already at 22 °C and, more clearly so, at 4 °C. This difference was also reflected in the RER, indicating a greater use of fat as the principal source of energy in the HFD groups. No differences among the individual groups were recorded in the heat or locomotor activity measurements. However, at 4 °C the heat value for pooled HFDs was significantly lower than in CD (p = 0.021), indicating higher energy expenditure in the latter. To verify the data obtained on VO_2_, VCO_2_ and heat by the conventional approach of normalizing the gas volumes to body weight, the data on individual groups were further assessed by ANCOVA with body weight as a covariate [38]; the outcomes were the same. Interestingly, the CD-fed mice had the highest calorie intake and drank the largest volume of water during the CLAMS measurements. Consequently, their body weight loss tended to be lower than in the HFD-fed groups (Fig. 6**G–I**), suggesting that HFD-feeding may have impaired the ability of the mice to adjust their energy metabolism with ambient conditions.

### 3.4 Histology

Liver and epiWAT samples of all mice were subjected to a histological analysis. In the liver, the type of lipid accumulation was assessed utilizing the Aiforia AI software [33] and inflammatory activity by counting inflammatory foci. In the epiWAT, adipocyte size and the number of CLS (consisting of Iba-1-expressing macrophages) was evaluated.

**Fig. 7:**
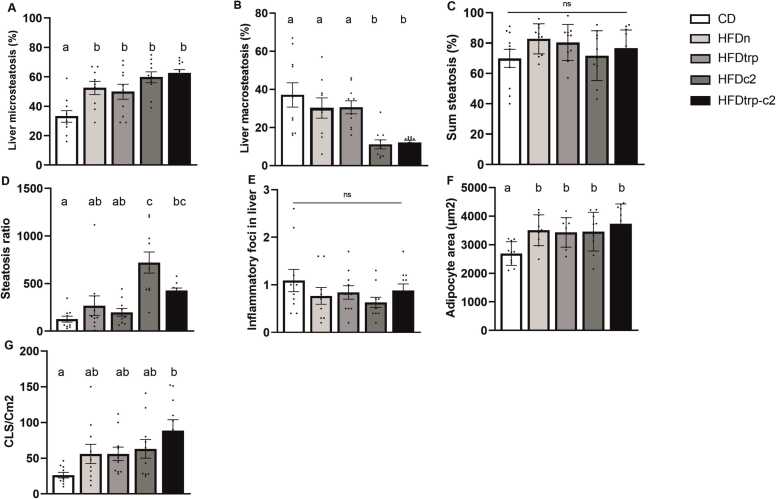
Quantitative histopathological effects of the diets in the liver and WAT. Hepatic micro- (A) and macrosteatosis (B), sum steatosis (C), steatosis ratio (micro/macro; D), and inflammatory foci (E). Adipocyte size (F) and CLS (F) in WAT. Columns with non-identical letters are statistically different at the significance level (p < 0.05). In panels C & E, “ns” denotes no significant difference among the groups (n = 10, means ± SEM).

**Fig. 8:**
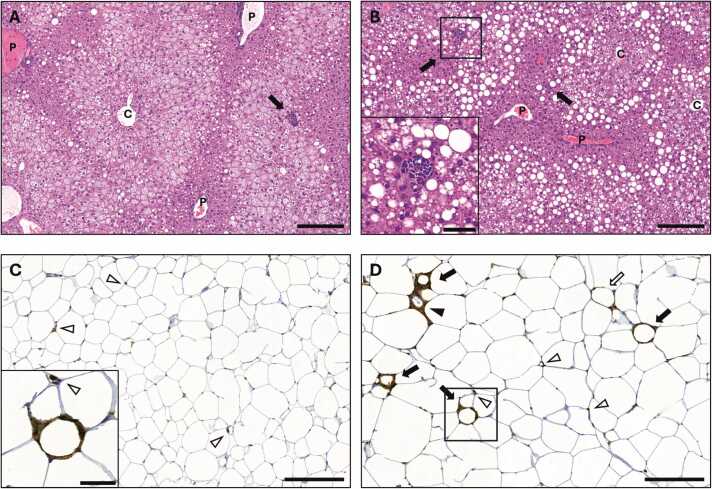
Micrographs captured from whole slide images exhibiting representative micro- and macrovesicular lipid accumulation and inflammatory foci in the liver (A, B; HE-stain), and adipocyte size and CLS in WAT (C, D; Anti Iba-1 antibody-immunohistochemistry). A. HFDtrp-c2 mouse liver: Microvesicular steatosis covers ∼ 70 % of the parenchymal area and occupies centrilobular and midzonal segments. Macrovesicular steatosis comprises ∼ 20 % of the parenchyme residing mainly between midzonal and periportal segments. A conspicuous central vein (C) and several portal veins (P) in portal triads are present as well as one medium-sized inflammatory focus (arrow). Bar 200 µm. B. HFDn mouse liver: Contradictory changes with 45 % macro- and ∼40 % microvesicular steatosis. The macrovesicular steatosis fills midzonal areas, while microvesicular steatosis restricts to centrilobular segments. One medium-sized (inset) and one small periportal inflammatory focus (arrows) are present. Central veins (C), portal veins (P). Bar 200 µm. Inset: Closeup of the medium-sized inflammatory focus consisting of macrophages, lymphocytes, neutrophils, and cell remnants. Bar 50 µm. C. CD mouse WAT: The median adipocyte area in the sample is ∼ 2800 µm^2^. Several brown, Iba-1-expressing macrophages (open arrowheads) but no CLS are present among adipocytes. Bar 200 µm. Inset: A CLS and a separate macrophage (open arrowhead) from micrograph D. Bar 50 µm. D. HFDtrp-c2 mouse WAT: The median adipocyte area in the sample is ∼ 4500 µm^2^. The section shows four CLS (arrows) and several macrophages (open arrowheads). Two structures reminiscent of, but not recorded as, CLS are present: macrophages encircle an interstitial capillary (arrowhead) and surround partly an adipocyte (open arrow). Bar 200 µm.

In the liver, the degrees of micro- and macrosteatosis in the test groups displayed virtually mirror images of each other: while the lowest percentages of microsteatosis were found in the CD group and the highest in the HFDc2 and HFDtrp-c2 groups, the converse was true for macrosteatosis (Figs. 7**A &** 7**B**). The HFDn and HFDtrp groups resembled the C2-treated groups for microsteatosis but CD for macrosteatosis. The sum of micro- and macrosteatosis percentages was similar in all five feeding groups indicating equivalent amounts of total fat (Fig. 7**C**). However, the ratio of micro- to macrosteatosis was higher in the two C2-supplemented groups than in the other three groups (Fig. 7**D**). The microvesicular form of steatosis was always located in the centrilobular area (in a few cases extending outside it), whereas macrovesicular steatosis predominantly localized to the midlobular region (Figs. 8**A &** 8**B**). There was a highly significant negative correlation between micro- and macrosteatosis (Table 1).

**Table 1:** Correlations among histopathological findings in the liver.

**Change**		**Foci**	**Micro**	**Macro**	**Total**^1^
**Foci**	Corr.coeff. (Spearman's rho)	1.000	0.326	− 0.45	− 0.154
	Sig. (2-tailed)		0.022	0.001	0.291
**Micro**	Corr.coeff. (Spearman's rho)	0.326	1.000	− 0.491	0.485
	Sig. (2-tailed)	0.022		< 0.001	< 0.001
**Macro**	Corr.coeff. (Spearman's rho)	− 0.45	− 0.491	1.000	0.433
	Sig. (2-tailed)	0.001	< 0.001		0.002
**Total**	Corr.coeff. (Spearman's rho)	− 0.154	0.485	0.433	1.000
	Sig. (2-tailed)	0.291	< 0.001	0.002	

1 Total steatosis (sum).

The severity of hepatic inflammation, as assessed by the number of inflammatory foci, did not differ among the groups (Fig. 7**E**). Intriguingly, inflammatory foci exhibited a significant positive correlation with microsteatosis but a highly significant negative correlation with macrosteatosis (Table 1). Hepatocellular ballooning, which associates with the progression of human NAFLD [34], was not detected.

In the epiWAT, all HFD-fed groups exhibited significantly larger adipocytes and more CLS (an index of inflammation) than the CD group (Figs. 7**F &** 7**G**). The correlation between WAT CLS and hepatic inflammatory foci was not significant (Spearman’s rho = − 0.254; p = 0.078). Thus, the HFDs induced a low-grade inflammation in the WAT but not in the liver. Representative photographs of histological changes in the liver and WAT in each group are shown in Suppl. Figs. S1 and S2.

### 3.5 Plasma biochemical analyses

**Fig. 9:**
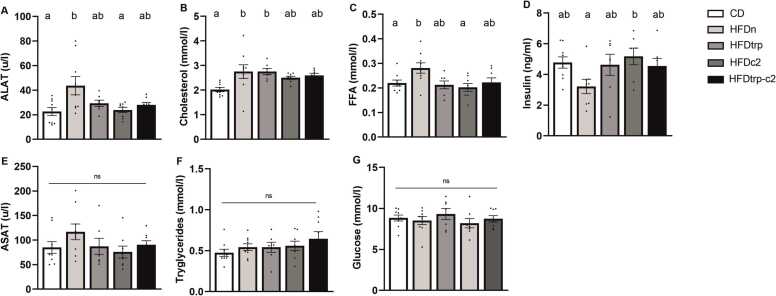
Effects of the diets on plasma ALAT (A), total cholesterol (B), free fatty acids (C), insulin (D), ASAT (E), triglycerides (F), and glucose (G). Columns with non-identical letters are statistically different at the significance level (p < 0.05). In panels E–G, “ns” denotes no significant difference among the groups (n = 10, means ± SEM).

All HFD groups tended to show higher total cholesterol values than CD (significant for HFDn and HFDtrp; Fig. 9**B**), and pooled HFDs differed from the CD group in a highly significant manner (p < 0.001). Statistically significant differences occurred sporadically also in ALAT, FFA and insulin, whereas no significant differences were observed in ASAT, triglycerides and glucose (Fig. 9**A–G**).

### 3.6 RT-qPCR analyses

**Fig. 10:**
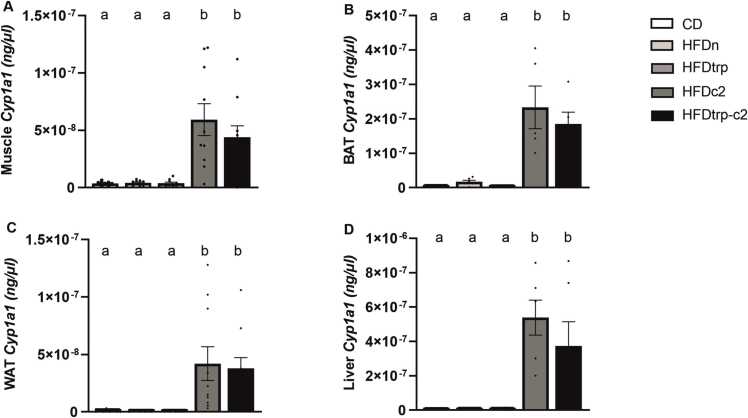
Expression of *Cyp1a1* in muscle (A), BAT (B), WAT (C), and liver (D). Columns with non-identical letters are statistically different at the significance level (p < 0.05) between the groups (n = 7–9, means ± SEM).

In the two groups treated with the AHR agonist C2, increased expression levels of *Cyp1a1*, a biomarker of AHR activation, were detected in all tissues analyzed (muscle, BAT, WAT, and liver; Fig. 10**A–D**). The mRNA abundance of *Fasn* (fatty acid synthase) displayed a downward trend in WAT and BAT of the HFD-fed mice, reaching significance for the HFDtrp group in BAT (Suppl. Figs. S3K & S3L). No statistically significant differences among the feeding groups were recorded in *Cpt1*, *Ucp1*, *Ucp2*, *Cd36, Fgf21, Ppara,* or *Pgc1a* in any of the tissues examined (Suppl. Figs. S3A–S3J & S3M).

## 4 Discussion

Modulation of AHR signaling in mice has been reported to result in discrepant effects on energy metabolism. Therefore, our study design included both activation (by C2 supplementation) and potential inactivation (through reduction of feed TRP) of AHR signaling. The core HFD feed we provided to our mice was moderately obesogenic (45 % of energy from fat) and was supplemented with 2 % cholesterol to render it more “Western diet-like” and to precisely match the diet which was reported to lead to substantial body weight gain in the C57Bl/6J mouse strain by Moyer et al. [11]. In contrast to the Moyer study, however, also our control diet contained 2 % cholesterol. Moreover, in the present study we used the physiologically more normal C57BL/6 JRccHsd mice instead of C57Bl/6J mice to enhance the relevance of the animal model.

When assessed by the body weight gain alone, the C57BL/6JRccHsd substrain appeared to be refractory to dietary obesity. In the face of twice as high a daily calorie intake compared with the CD-fed mice, the HFD-fed groups gained weight only 28–38 % more than the controls on the CD. However, this is probably due to the fact that the density of mammalian skeletal muscle is considerably greater than that of adipose tissue [39], [40], because differences in neither energy expenditure nor motility could explain the discrepancy. Indeed, the relative weight of epididymal fat was 43–61 % higher in the HFD groups than in CD-fed mice. It has been established previously that the gonadal fat deposit represents an excellent estimate of the total bodily fat in mice, achieving the same prediction accuracy as the dissection of all four fat depots [41]. Hence, despite their relatively small acceleration of body weight gain, the HFD-fed mice still underwent a change in body composition typical of obesity.

In our experimental setting, neither supplementation of the HFD with C2 nor reduction of its TRP concentration proved to cause any major impacts on body weight gain, fat accumulation, glucose tolerance or insulin sensitivity. Insufficient dosage of C2 seems unlikely because *Cyp1a1* induction, a broadly-applied index of AHR activity, was clearly discernible at termination in all tissues examined. In previous studies, activation of the AHR/CYP1A1 pathway has been reported to exert either diabetogenic or antidiabetogenic effects [42]. Likewise, opposite impacts on body weight gain have also been found [18], [19].

As to the TRP restriction, a recent study by Zapata et al. [24] reported that providing obesity-prone rats a HFD (40 % of energy from fat) containing 70 % of the TRP requirement induced sympathetically-mediated thermogenesis without affecting body composition. Since a large number of dietary and endogeneous AHR ligands are TRP derivatives (including FICZ), we hypothesized this to be due to diminished AHR activity. We further contemplated that by increasing energy expenditure, lowered AHR signaling would enable the animal to meet its protein needs from low protein–high energy nutrients without getting fat. Unfortunately, in the present study the basal expression level of *Cyp1a1* was so low in all the tissues examined that the sensitivity of our RT-qPCR system did not suffice to reveal whether the expression level was diminished in the HFDtrp group. Nevertheless, calorie intake was slightly but significantly higher in the two low-TRP groups concomitant with equal body weight change and body fat content (inferred from epididymal fat) in comparison with their respective full-TRP groups. This suggests that low-TRP feeding may induce energy expenditure also in mice. However, the CLAMS or gene expression data did not support that conclusion. Another alternative is that a reduction in TRP may impair energy absorption by altering the intestinal composition of microbes or their metabolites [43]. Therefore, further studies on this phenomenon are clearly warranted.

In hepatic histology, a high proportion of steatotic areas (∼ 70 % in total) was recorded in the CD group, probably due to the cholesterol added also into this feed. The fact that total steatosis did not differ among the groups attests to cholesterol being a major driver of this change, as reported earlier [44], [45]. However, the type of hepatic fat accumulation did vary, with the C2-supplemented groups standing out from all others in this respect, as assessed by a recently developed AI methodology for the analysis [33]. In all HFD-fed groups, microsteatosis was augmented in comparison with CD-fed mice (most in C2-supplemented groups). In contrast, macrosteatosis remained at the CD group level in HFDn and HFDtrp groups, but was reduced by almost 70 % in both HFDc2 and HFDtrp-c2 groups, leading to a strikingly elevated micro-/macrosteatosis ratio. To the best of our knowledge, this drastic shift has not been associated with AHR activation earlier, and overall, authors have previously given little attention to the histological type of hepatic steatosis. However, the qualitative distinction is of considerable importance because the two types of steatosis emanate from different processes and represent conditions of widely different severity. In macrosteatosis, the principal type of steatosis in humans, the hepatocyte contains a single large vacuole of fat, which displaces the nucleus to the periphery of the cell. In the early phases (before progression towards steatohepatitis), it is usually a fairly benign change and associated with such disorders as obesity, diabetes and alcohol abuse. In microvesicular steatosis, by contrast, the hepatocyte is expanded by numerous small lipid vesicles, which leave the nucleus in the center of the cell. Microsteatosis is a more severe condition than macrosteatosis and will arise whenever there is severe impairment of mitochondrial β-oxidation [46], [47], [48]. If prolonged, especially micro- but also macrosteatosis can progress to steatohepatitis as excess of FFA will overload the capacity of the electron transfer chain in mitochondria causing oxidative stress and lipid peroxidation [47], [49].

Since AHR agonists can induce oxidative stress in the liver [50], the increased ratio of micro-/macrosteatosis by C2 may indicate that the transition from steatosis to steatohepatitis could be accelerated in these mice. Supporting this contention, hepatic lipid peroxidation is exacerbated in transgenic mice with constitutively active AHR [51]. Moreover, the transition of steatotic HepaRG cells exposed to benzo[*a*]pyrene and ethanol to a steatohepatitis-like state was demonstrated to be dependent on AHR activation and attributable to mitochondrial dysfunction and ROS generation [52]. On the other hand, AHR activation can also confer protection from oxidative stress by inducing *Nrf2* expression [53]. In addition, dietary AHR agonists will bring about AHR activation in intestinal intraepithelial cells, which augments barrier integrity, enhances the production of antimicrobial peptides, and modifies the intestinal microbiome. Thereby, they may reduce inflammation and attenuate steatohepatitis of different origins [54], [55], [56], [57].

In the present study, no evidence of impaired mitochondrial function in the C2 groups was recorded by CLAMS. Furthermore, the degree of hepatic inflammation was similar in all four HFD-fed groups, ALAT activity was low, and the hepatic expression of *Cpt1*, which encodes the rate-limiting enzyme of fatty acid oxidation, carnitine palmitoyltransferase 1A, was at a similar level in all experimental groups including those treated with C2. Likewise, the expression of the peroxisome proliferator-activated receptor γ co-activator 1α gene (*Pgc1a*) did not differ among the groups. PGC1α is a key regulator of mitochondrial quality control mechanisms [58]. The predominant impact of C2 was reduction of macrosteatosis rather than induction of microsteatosis, thereby suggesting an alteration in lipid metabolism compared with the effect of unsupplemented HFD. However, little support for this was found, although plasma cholesterol concentration was slightly lower in the C2-supplemented groups than in the two other HFD groups, but without statistical significance. Thus, the pathogenesis of the altered type of hepatic lipid accumulation by C2 treatment as well as its biological significance remain to be established.

The strengths of the present study are a long exposure time, inclusion of both AHR induction and potential reduction of AHR activity through TRP restriction, and a broad array of energy balance-related variables measured. The main limitation of this study is that it was conducted in male mice alone. This was due to both economical reasons and the fact that a great preponderance of previous studies on energy metabolism have employed only males. It is well established that in mice sex can influence a number of the variables determined here. However, regarding the major ones of interest, males could be expected to represent a more responsive model. For example, on HFD male mice usually gain more weight, are more prone to hepatic steatosis, and develop more severe glucose intolerance and insulin resistance than females [59], [60], [61]. Moreover, male mice are more susceptible to the AHR-mediated acute toxicity of TCDD than their female counterparts [62]. Therefore, it is not likely that in female mice the responses would have been more prominent, but this contention needs to be verified by in vivo experiments.

To conclude, in this study a long-term feeding of male C57BL/6 JRccHsd mice with cholesterol-enriched HFD (45 % of energy from fat) doubled calorie consumption from that on cholesterol-enriched CD. It further increased visceral fat, adipocyte size, and WAT inflammation, but enhanced body weight gain only by 28–38 % and did not affect the degree of total liver steatosis. Reduction of the TRP concentration of this HFD augmented calorie intake further while concurrently not accelerating body weight gain. Supplementation of the HFD with the AHR agonist C2 led to a change in the type of hepatic fat accumulation, mitigating macrosteatosis while tending to promote microsteatosis. Reconciling these findings with previously published data suggest that the effect of AHR activation on energy balance is context- (type of AHR activation [ligand vs. non-ligand]; ligand specificity; duration, breadth, and degree of activation) and model-dependent (species and strain). Low dietary TRP concentration, in turn, may enhance energy expenditure, but either this impact is more pronounced in rats than in mice or it varies among mouse (sub)strains.

## Author´s statement

All authors have read and approved the final manuscript. The authors declare no competing interests. This work has not been published elsewhere and is not under consideration by another journal. Raimo Pohjanvirta reports financial support from the Academy of Finland (Grant #338433). All the other authors declare that they have no known competing financial interests or personal relationships that could have appeared to influence the work reported in this paper. All experiments were authorized by the National Animal Experiment Board in Finland (Eläinkoelautakunta, ELLA, license code ESAVI/45477/2019). All procedures were conducted in accordance with Directive 2010/63/EU of the European Parliament and Council.

## CRediT authorship contribution statement

**Avinash Bathina:** Writing – original draft, Visualization, Investigation, Formal analysis. **Atso Raasmaja:** Writing – review & editing, Investigation. **Janne Hakanen:** Writing – review & editing, Investigation, Formal analysis. **Laura Mairinoja:** Writing – review & editing, Software, Formal analysis. **Jere Lindén:** Writing – review & editing, Investigation, Formal analysis. **Suraj Unniappan:** Writing – review & editing, Formal analysis. **Raimo Pohjanvirta:** Writing – review & editing, Visualization, Validation, Supervision, Resources, Project administration, Methodology, Investigation, Funding acquisition, Formal analysis, Conceptualization. **Lars Pettersson:** Writing – review & editing, Resources.

## Funding

The study was supported by a grant to R.P. from the 10.13039/501100002341Academy of Finland (#338433).

## Declaration of Competing Interest

The authors declare the following financial interests/personal relationships which may be considered as potential competing interests: Raimo Pohjanvirta reports financial support from the Academy of Finland. All the other authors declare that they have no known competing financial interests or personal relationships that could have appeared to influence the work reported in this paper.

## Data Availability

Data will be made available on request.
