# Supplementary material for: Modulation of the effects of a cholesterol-supplemented high-fat diet by aryl hydrocarbon receptor (AHR) activation and/or tryptophan reduction in male mice
Source: Toxicol Rep. 2025 Jul 7;15:102083. doi: 10.1016/j.toxrep.2025.102083 (PMC12274841; doi:10.1016/j.toxrep.2025.102083)
Supplement: Supplementary file 1 — Supplementary material [file mmc1.docx]

**Supplemental Table S1. Diet compositions**

|  | **Control diet**  **(CD)** | **High-fat diet (HFDn)** | **Reduced tryptophan diet**  **(HFDtrp)** |
| --- | --- | --- | --- |
| **Ingredient** | **gm** | **gm** | **gm** |
| Casein | 0 | 0 | 0 |
| L-Cystine | 4.2 | 4.2 | 4.2 |
| L-Isoleucine | 7.6 | 7.6 | 7.6 |
| L-Leucine | 15.8 | 15.8 | 15.8 |
| L-Lysine | 13.2 | 13.2 | 13.2 |
| L-Methionine | 5.1 | 5.1 | 5.1 |
| L-Phenylalanine | 8.4 | 8.4 | 8.4 |
| L-Threonine | 7.2 | 7.2 | 7.2 |
| L-Tryptophan | 2.1 | 2.1 | 1.47 |
| L-Valine | 9.3 | 9.3 | 9.3 |
| L-Histidine | 4.6 | 4.6 | 4.6 |
| L-Alanine | 5.1 | 5.1 | 5.1 |
| L-Arginine | 6.0 | 6.0 | 6.0 |
| L-Aspartic Acid | 12.1 | 12.1 | 12.1 |
| L-Glutamic Acid | 38.2 | 38.2 | 38.2 |
| Glycine | 3.0 | 3.0 | 3.0 |
| L-Proline | 17.8 | 17.8 | 17.8 |
| L-Serine | 10.0 | 10.0 | 10.0 |
| L-Tyrosine | 9.2 | 9.2 | 9.2 |
| Corn starch | 452.2 | 72.8 | 73.55 |
| Maltodextrin 10 | 75 | 100 | 100 |
| Sucrose | 172.8 | 172.8 | 172.8 |
| Cellulose, BW200 | 50 | 50 | 50 |
| Soybean Oil | 25 | 25 | 25 |
| Lard | 20 | 177.5 | 177.5 |
| Mineral Mix S10026 | 10 | 10 | 10 |
| DiCalcium Phosphate | 13 | 13 | 13 |
| Calcium Carbonate | 5.5 | 5.5 | 5.5 |
| Potassium Citrate, 1 H2O | 16.5 | 16.5 | 16.5 |
| Sodium Bicarbonate | 7.5 | 7.5 | 7.5 |
| Vitamin Mix V10001 | 10 | 10 | 10 |
| Choline Bitartrate | 2 | 2 | 2 |
| Cholesterol | 18 | 18 | 18 |
| Total | 1056.45 | 859.55 | 859,67 |

| **Kcal%** | | | |
| --- | --- | --- | --- |
|  | **Control diet** | **High fat diet** | **Reduced tryptophan diet** |
| Protein | 18 | 18 | 18 |
| Carbohydrate | 71 | 35 | 35 |
| Fat | 10 | 46 | 46 |
| Total | 100 | 100 | 100 |
| Kcal/gm | 3.7 | 4.6 | 4.6 |
| Tryptophan (g/kg) | 1,99 | 2,44 | 1,71 |

**Supplemental Table S2. Sequences of the primers used**

**Gene Forward primer** (5’–3’) **Reverse primer** (5’–3’)

*Cyp1a1* TCACGATTGTTTTGGACCTCT GCCAATCACTGTGTCTAGTTCC

*Ucp1* ATCAACTCTCTGCCAGGACA AGAAAAGAAGCCACAAACCCTT

*Ucp2* TTGGCCTCTACGACTCTGTC CACATCTGTAGGCTGGGCTA

*Ppara* CAACATGAACAAGGTCAAGGC AAGCGTCTTCTCGGCCATAC

*Cd36* CTTCCACATTTCCTACATGCAAG TGCAAATTGTAGAGTGAATCCAG

*Fasn* CCTACACCCAGAGCTACCG CTTCCAGGCTCTTCAGTGG

*Fgf21* GCCAGGGGTCATTCAAATCC TCAGGATCAAAGTGAGGCGAT


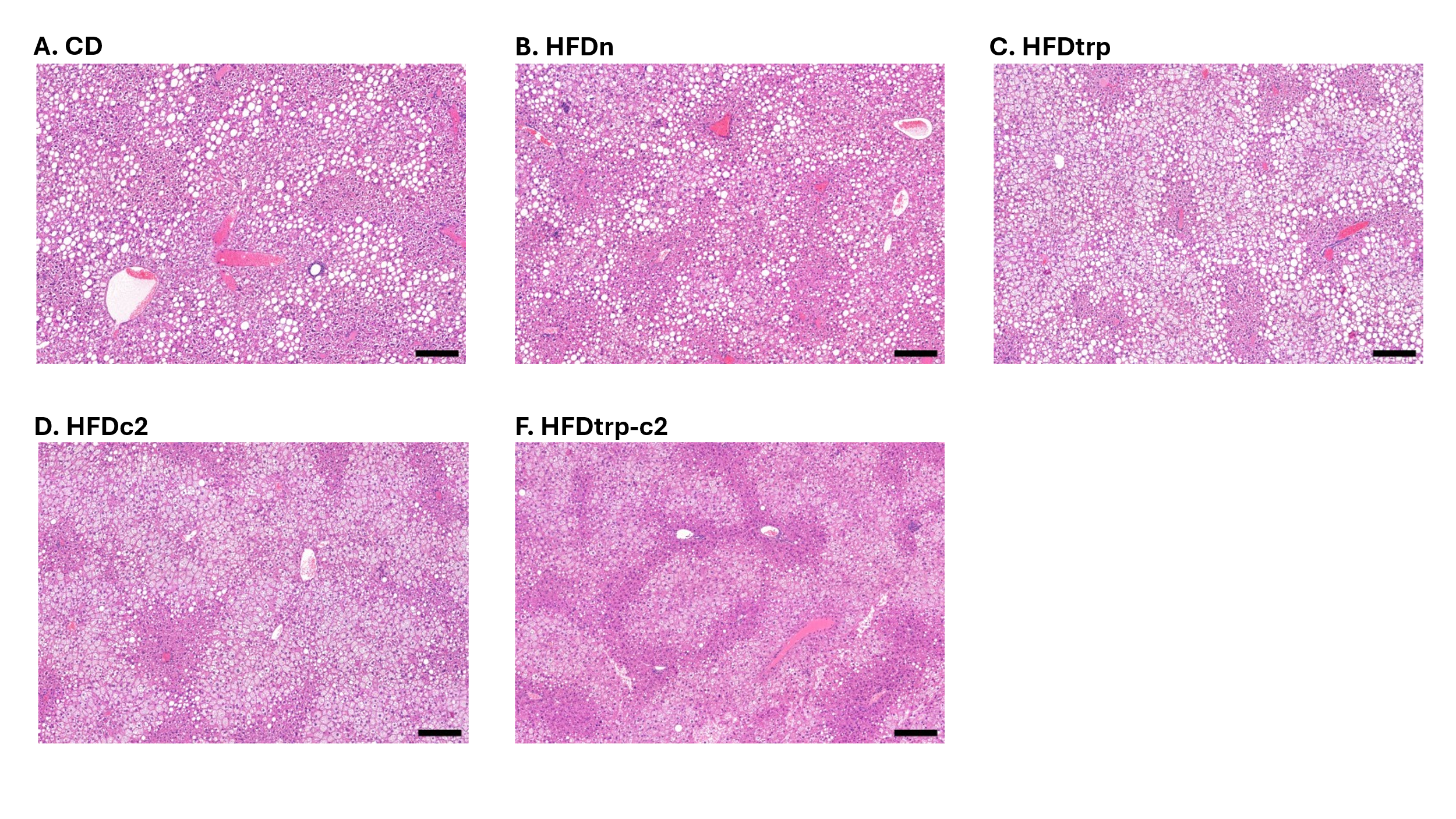
**Supplemental Fig. S1**. Representative images of hepatic histology in each group


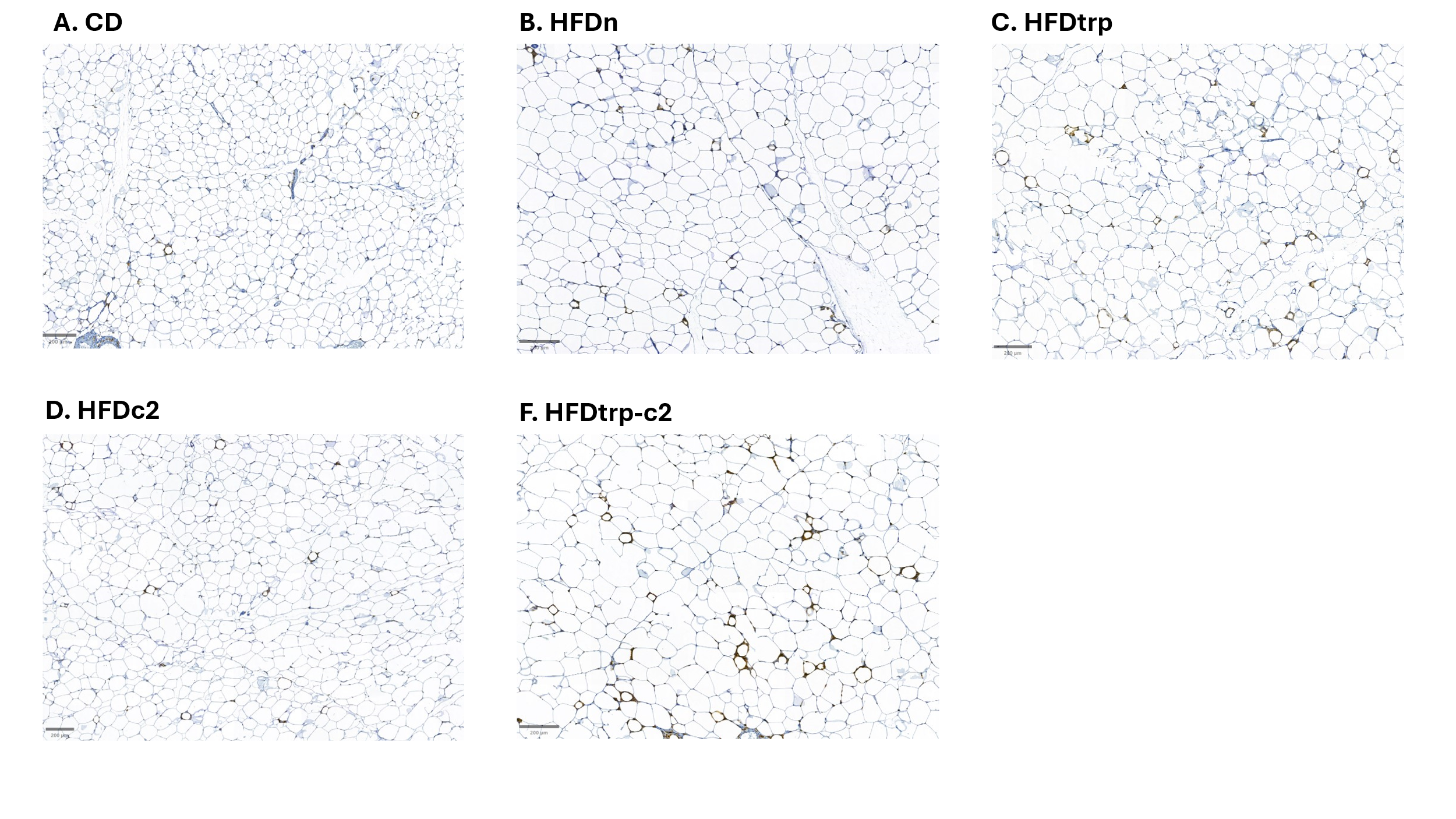


**Supplemental Fig. S2.** Representative images of WAT histology in each group


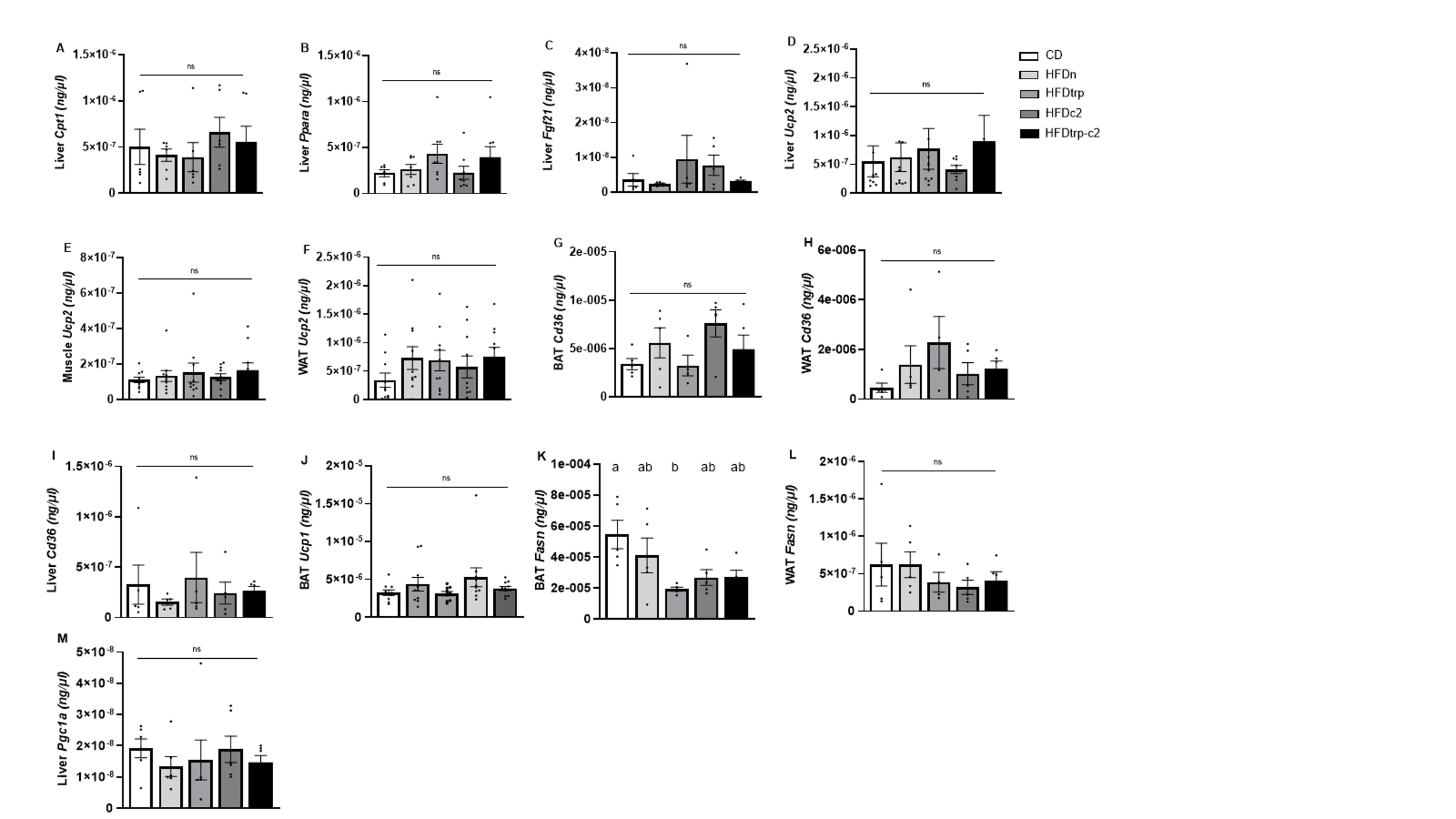


**Supplemental Fig. S3.** Expression of liver *Cpt1*(A), liver *Ppara* (B), liver *Fgf21* (C), liver *Ucp2* (D), muscle *Ucp2* (E), WAT *Ucp2* (F), BAT *Cd36* (G), WAT *Cd36* (H), liver *Cd36* (I), BAT *Ucp1* (J), BAT *Fasn* (K), WAT *Fasn* (L), and liver *Pgc1a* (M). In panel K, columns with non-identical letters are statistically different at the significance level (p < 0.05). In other panels, “ns” denotes no significant difference among the groups (n=7-9, means ± SEM).
